# Psychosocial Evaluation of Adults with Primary Immunodeficiency

**DOI:** 10.1007/s10875-024-01671-3

**Published:** 2024-02-27

**Authors:** Reyhan Gumusburun, Sevgi Altay, Hasancan Cengiz, Gulendam Hakverdioglu Yont, Ozlem Kuman Tuncel, Omur Ardeniz

**Affiliations:** 1 Department of Internal Medicine, Division of Allergy and Clinical Immunology, Ege University Medical Faculty, Izmir, Turkey; 2 Incesu State Hospital, Kayseri, Turkey; 3 Nursery Department, Health Science Faculty of Izmir Tınaztepe University, Izmir, Turkey; 4 Psychiatry Department of Ege University Hospital, Izmir, Turkey

**Keywords:** Immunodeficiency, Anxiety, Depression, Loneliness, Psychometric scale

## Abstract

**Purpose:**

Primary immunodeficiency disorder (PID) is a heterogeneous group of diseases characterized by immune dysregulation and increased susceptibility to infections, with various cognitive, emotional, behavioral, and social effects on patients. This study aimed to evaluate loneliness, social adaptation, anxiety, and depression and to identify associated factors in adults with immunodeficiency.

**Methods:**

A cross-sectional study in Turkey (Feb-Aug 2022) obtained sociodemographic data from patient records. The Social Adaptation Self-Evaluation Scale (SASS), UCLA-Loneliness Scale (UCLA-LS), and Hospital Anxiety and Depression Scale (HADS) were administered in individual patient interviews. HADS-Anxiety (HADS-A) and HADS-Depression (HADS-D) scores were assessed using cut-offs of 10 and 7, respectively; SASS cut-offs for social imbalance and normalcy were < 25 and > 35, respectively.

**Results:**

A total of 104 patients (60 women, 44 men) with a median age of 34 years (range: 18–89) were included in the study. Mean scores were SASS: 34.46 ± 8.11, UCLA-LS: 44.89 ± 12.66, HADS-A: 9.87 ± 4.77, and HADS-D: 9.12 ± 4.80. SASS score was negatively correlated with HADS-A, HADS-D, and UCLA-LS scores. There were positive correlations between UCLA-LS and HADS-A (r = -0.355, p < 0.01) and HADS-D (r = -0.614, p < 0.01) and between HADS-A and HADS-D (r = -0.454, p < 0.01). Low-income level was associated with higher HADS-A, HADS-D, and UCLA-LS scores and lower SASS score (p = 0.012, p = 0.041, p = 0.008, and p = 0.001, respectively).

**Conclusion:**

Adults with PID are at risk for depression and experience high levels of loneliness. Social maladjustment and loneliness contribute to anxiety and depression, and loneliness is correlated with impaired social functioning. These findings emphasize the importance of biopsychosocial evaluation of individuals diagnosed with PID.

## Introduction

Primary immunodeficiency (PID) may manifest as recurrent, severe, atypical infections or autoimmunity, lymphoproliferation, or malignancies at diagnosis or during the course of the disease. There are many different types of PIDs, ranging from milder forms that are diagnosed at older ages to life-threatening forms with infancy onset. Although PID is better recognized now, establishing a correct diagnosis is often challenging due to the heterogeneous clinical and immunological phenotypes. The more widespread use of new genetic testing techniques and equipment has revealed a growing number of genetic defects in PID patients. According to the latest data, 485 PIDs have been genetically identified [1]. Conventional treatment for PID is immunoglobulin replacement therapy (IGRT), which can be administered intravenously (IVIG) or subcutaneously (SCIG). IGRT effectively prevents infections and related complications, thereby reducing the number of deaths from infectious diseases. Genetic, molecular, cellular, and immunological advances have provided a greater understanding of the pathogenesis and enabled mechanism-based treatment approaches. Both early diagnosis and new treatment modalities are allowing people with PID to live longer. As a result, family physicians need to focus more on PID patients’ treatment adherence, care needs, psychological well-being, and quality of life.

Individuals with PID may develop neuroimmunopsychiatric disorders due to the direct pathophysiological effects of the physical illness itself. They are also susceptible to psychiatric disorders, loneliness, and social dysfunction because of disease perceptions and its impact on life. Few studies have investigated the prevalence and symptoms of psychiatric disorders and psychiatric diagnoses in this patient group. In a 2020 population-based cohort study investigating the relationship between psychiatric disorders and suicidal behavior, patients with PID were found to have higher rates of suicide attempts, suicide, and various psychiatric disorders compared to the healthy population. When individuals with and without PID were compared, the strongest association with an individual psychiatric disorder was with autism spectrum disorders (1.1% of all individuals with PID), followed by eating disorders (0.8%), anxiety disorders (9.3%), obsessive–compulsive disorder (0.6%), major depressive and other mood disorders (10.4%), attention- deficit/hyperactivity disorder (1.5%), bipolar disorder (1.2%), substance abuse (6.1%), and schizophrenia and other psychotic disorders (2.4%), respectively [2]. In another study in 2022, 174 patients with PID were screened using a survey assessing distress, depression, anxiety, and somatization symptoms in comparison to the general population. PID patients scored significantly higher in all four dimensions [3].

Social functioning is the ability to interact with other people and fulfill a social role [4]. Social adjustment is impaired in many psychiatric and neurological diseases [5]. For instance, social dysfunction is more frequently co-occurring in depressive disorders compared to anxiety disorders. However, it becomes more pronounced in situations where anxiety and depression coexist [6]. Loneliness is a poignant, subjective, and emotional state characterized by the perceived disparity between desired and actual patterns of social interaction[7]. Research on loneliness has shown that it can cause both psychiatric disorders such as personality disorders, schizophrenia, suicidal ideation, depression, alcohol abuse, and sleep problems, as well as physical conditions such as immune dysregulation, metabolic syndrome, and cardiovascular disease [8, 9].

Previous research has indicated that PID patients are often at risk for anxiety and depression [3, 10, 11]. However, it is noteworthy that the dimensions of loneliness and social adaptation have not been scrutinized within this specific patient cohort. Additionally, recent studies have emphasized that loneliness and social adjustment may serve as noteworthy early indicators of depression. [6, 12, 13]. Therefore, the objective of this study is to assess the prevalence of anxiety, depression, loneliness, and social adjustment among adults diagnosed with PID, utilizing the Social Adaptation Self-Evaluation Scale (SASS), UCLA-Loneliness Scale (UCLA-LS), and Hospital Anxiety and Depression Scale (HADS). Simultaneously, the study aims to identify socio-demographic and socio-economic characteristics, as well as factors related to the disease and treatment process that may influence these conditions. Furthermore, the research endeavors to analyze the relationships between SASS, UCLA-LS, and HADS.

## Materials and Methods

### Participants and Study Design

**Fig. 1:**
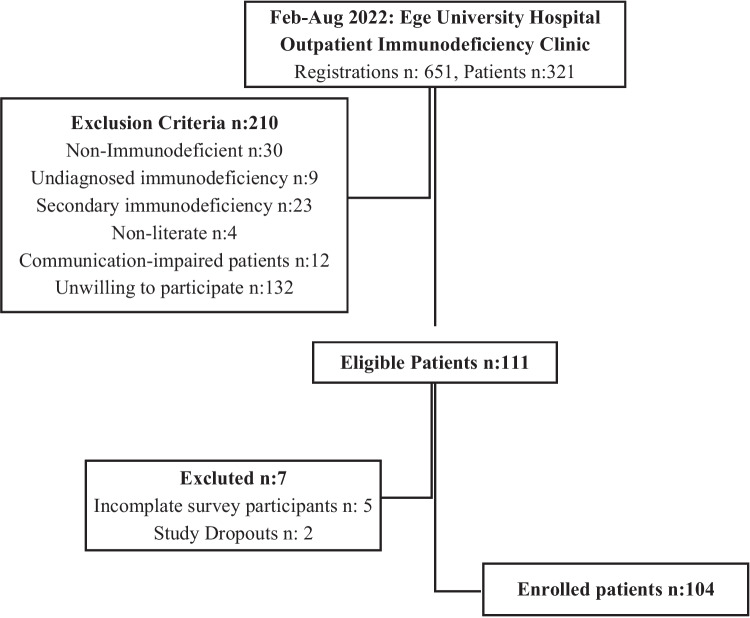
Inclusion Flowchart

The cross-sectional study was conducted in the adut Immunology and Allergy Department of Ege University Hospital, which functions as a tertiary referral center. The study population consists of individuals aged 18 and above who presented to the outpatient Immunology Clinic between February and August 2022, diagnosed with PID according to international guidelines [1, 14–16]. No sampling was conducted, and the entire population was used as the sample (Fig. 1). Non-immunodeficient, undiagnosed immunodeficiency, secondary immunodeficiency, and patients with no Turkish literacy or communication skills were not included in the study. The study protocol was approved by the ethics committee of the Izmir Tınaztepe University School of Medicine. After providing information about the study and obtaining written consent from all participants, they were asked to complete the SASS, UCLA-LS, HADS, and Case Report Form. The average time for survey completion ranged between 20–30 min. Assistance was provided to those facing difficulties in completing the questionnaires. Subsequently, data were evaluated through a single session of face-to-face individual patient interviews, lasting approximately 30 min, conducted by an allergy and immunology specialist.

### Data Collection Instruments

*Case Report Form:* This form has been developed by the researchers. It includes socio-demographic and socio-economic characteristics such as age, gender, height, weight, place of residence (village, small town, city), family type (nuclear, extended), marital status (single, married, divorced), education (none, primary, secondary, university), employment status (working, retired, students, unemployed), income level (low, adequate, high), presence of siblings (yes, no), number of siblings, parental consanguinity (yes, no), smoking and alcohol consumption status (yes, no), presence of disability report application (yes, no), acceptance of disability report application (yes, no), mode of transportation to the hospital (public transport, private vehicle). Income level was classified based on a subjective self-report. The patients were asked to rate their income level as low (if their income was less than their expenses), adequate (income covers expenses), or high (income is more than expenses). Additionally, it covers disease-related characteristics such as the age of symptom onset for PID, age at PID diagnosis, presence of accompanying comorbidities (yes, no), diagnosed psychiatric illness (yes, no), family history of psychiatric illness (yes, no), family history of death in early infancy (yes, no), family history of PID (yes, no), history of hospitalization due to immunodeficiency (yes, no), length of hospital stay (days), and treatment-related features like immunoglobulin treatment use (yes, no), immunoglobulin treatment modality (IVIG, SCIG, facilitated SCIG (fSCIG)), and perceived quality of immunoglobulin replacement therapy (IGRT) (very bad/bad, moderate, good/very good).

*Social Adaptation Self-Evaluation Scale (SASS):* The SASS measures social motivations and behaviors. It covers various areas of social functioning, including work, leisure, family, environmental organization and coping skills. The scale items are scored from 0 to 3. The SASS has 21 items, but only one of the first two items is scored depending on whether or not the respondent has an occupation. Therefore, the total score is the sum of 20 items and ranges between 0 and 60. A score of 35 or higher is interpreted as normal social functioning, while scores below 25 indicate problematic social functioning. In the validity and reliability study of the Turkish version of the SASS conducted by Akkaya et al. in 2008, it was found to have a Cronbach’s alpha internal consistency coefficient of 0.90, item-total score correlations of 0.22–0.66, and a test–retest correlation coefficient of 0.770. [17].

*Hospital Anxiety and Depression Scale (HADS):* The scale consists of 14 items, 7 of which measure anxiety (HADS-A) and 7 of which measure depression (HADS-D). The items are rated from 0 to 3, yielding a total score of 0–21 for each subscale, with higher scores indicating more severe symptoms. The Turkish adaptation of the HADS was conducted by Aydemir et al. in 1997. In the Turkish validation study, the cut-off points were determined as 10 for the HADS-A and 7 for HADS-D [18].

*UCLA Loneliness Scale (UCLA-LS):* The scale consists of ten questions measuring satisfaction in social relationships and ten questions measuring dissatisfaction, totaling 20 items. The items are rated between 1 and 4, for a total score ranging from 20 to 80. Higher scores indicate stronger feelings of loneliness. The Turkish validation of the UCLA-LS was carried out by Demir in 1989. The Cronbach’s alpha coefficient of the scale is 0.96, and the test–retest reliability coefficient was 0.942 [19].

### Statistical Analysis

The frequencies and percentages were given for categorical variables and, mean, standard deviation (SD), minimum and maximum values for numerical variables as descriptive statistics. Association between two categorical variables were analyzed with Pearson’s chi-squared test or the Fisher’s exact test. Shapiro Wilk test was used to check normality assumption. Independent samples t-test or Mann Whitney U test was performed to compare numerical variables between two groups. The analysis of variance (ANOVA) or Kruskal Wallis test were chosen to compare three or more groups. Pearson correlation coefficient was used to evaluate linear relationship between numerical variables. Statistical significance was assessed at p < 0.05 and all statistical analyses were performed using IBM SPSS Statistics 25.0 (IBM SPSS Statistics for Windows, Version 25.0. Armonk, NY: IBM Corp.)

## Results

**Fig. 2:**
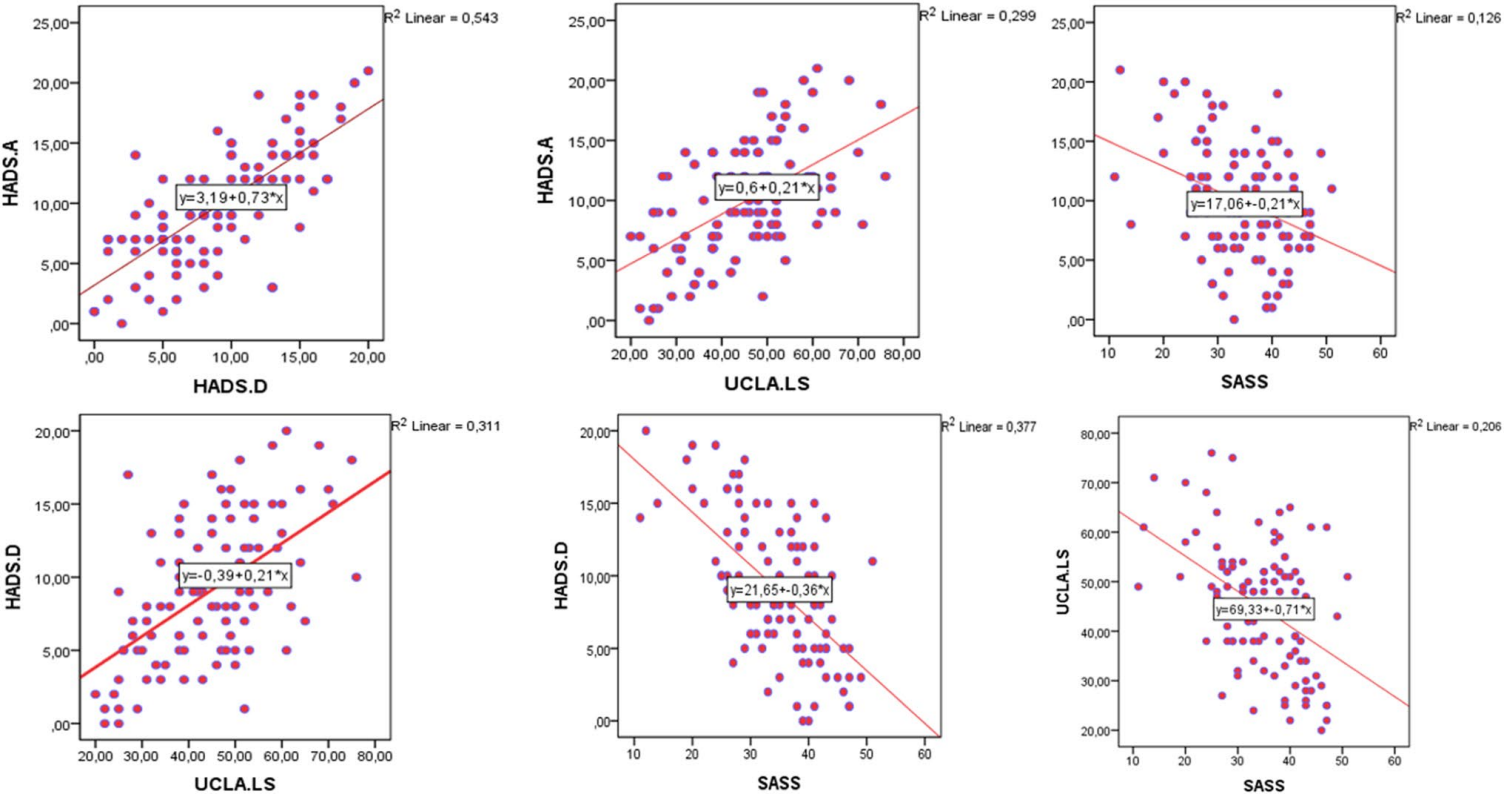
Correlations among scales

The sociodemographic data of the 104 participants in this study are presented in Table 1. Nearly all patients (96.2%) were receiving IGRT, and the patients’ infusion characteristics, treatment satisfaction, and hospitalization history are summarized in Table 2. The mean scale scores were 34.46 ± 8.11 for SASS, 44.89 ± 12.66 for UCLA-LS, 9.87 ± 4.77 for HADS-A, and 9.12 ± 4.80 for HADS-D. HADS-A score was higher than 10 in 46 patients (44.2%) and HADS-D score was higher than 7 in 64 patients (61.5%). On the SASS, nine patients (8.7%) scored below 25 and 50 patients (48.1%) scored above 35. SASS score was negatively correlated with HADS-A score (r = -0.355, p < 0.01), HADS-D score (r = -0.614. p < 0.01), and UCLA-LS score (r = -0.454, p < 0.01) (Fig. 2). Moreover, UCLA-LS was positively correlated with HADS-A (r = 0.547, p < 0.01) and HADS-D (r = 0.558, p < 0.01) and there was a positive correlation between HADS-A and HADS-D scores (r = 0.737, p < 0.01). Associations between sociodemographic characteristics and SASS, HADS-A, HADS-D, and UCLA-LS scores are presented in Table 3.

**Table 1:** Sociodemographic data of the study group (N = 104)

Variables		Variables	
Female, n (%)	60 (57.7%)	Age at diagnosis (years), median (range)	27 (1–71)
Age (years), median (range)	34 (18–89)	Age at symptom onset (years), median (range)	19 (1–71)
BMI, median (range)	22.66 (13.3–42.1)	Comorbidity n (%)	72 (69.2%)
Diagnosed psychiatric illness n (%)	27 (26%)	Alcohol, n (%)	10 (9.8%)
		Smoking, n (%)	7 (6.8%)
Siblings n (%)	96 (92%)	Family history of PID, n (%)	13 (12.5%)
Number of siblings, median (range)	2 (0–11)	Family history of death in early infancy, n (%)	33 (31.7%)
Parental consanguinity, n (%)	30 (28.8%)	Family history of psychiatric illness, n(%)	8 (7.7%)
Education, n (%)		Marital status, n (%)	
None	3 (2.9%)	Single	35 (33.7%)
Primary	16 (15.4%)	Married	58 (55.8%)
Secondary	32 (30.8%)	Divorced	11 (10.6%)
University	53 (51.0%)		
Employment status, n (%)		Place of residence, n (%)	
Working	36 (34.6%)	Village	25 (24.0%)
Retired	16 (15.4%)	Small town	41 (39.4%)
Students	7 (6.7%)	City	38 (36.5%)
Unemployed	45 (43.3%)		
Income level, n (%)		Family type, n (%)	
Low	41 (39.4%)	Nuclear	98 (94.2%)
Adequate	55 (52.9%)	Extended	6 (5.8%)
High	8 (7.7%)		
Applied for a disability report, n (%)	50 (48.5%)	Transportation, n (%)	59 (57.3%)
Yes	31 (29.8%)	Public transport	44 (42.7%)
Accepted		Private vehicle

**Table 2:** Patient characteristics by IGRT modality and hospitalization

IGRT, n (%)	
IVIG	76 (73.1%)
SCIG	21 (20.2%)
fSCIG	3 (2.9%)
None	4 (3.8%)
Satisfaction with IGRT, n (%)	
Yes	85 (85%)
IVIG	67 (88%)
SCIG	16 (76.2%)
fSCIG	2 (66.7%)
No	15 (15%)
Perceived IGRT quality, n (%)	
Very bad/ bad	5 (5%)
Moderate	27 (27%)
Good/ very good	68 (68%)
Hospitalization, n (%)	79 (76%)
Length of hospital stay (days), median (range)	20 (1–180)

IGRT: Immunoglobulin replacement therapy, IVIG: Intravenous immunoglobulin, SCIG: Subcutaneous immunoglobulin, fSCIG: Facilitated SCIG

**Table 3:** Association between sociodemographic characteristics and SASS, HADS-A, HADS-D, and UCLA-LS scores

Variables	n	HADS-A			HADS-D			UCLA-LS			SASS		
		Mean	SD	p	Mean	SD	p	Mean	SD	p	Mean	SD	p
Family history of PID	Yes: 13 No: 91	11.62 9.6	4.42 4.79	0.159	9.23 9.10	4.51 4.87	0.927	**51.62** **43.93**	**9.80** **12.78**	**0.04**	33.62 34.58	7.74 8.20	0.690
Comorbidity	Yes: 72	10.36	4.81	0.113	9.69	4.76	0.065	**45.94**	**12.33**	**0.020**	33.58	7.80	0.098
	No: 32	8.75	4.57		7.81	4.71		**42.53**	**13.27**		36.44	8.57	
Family history of psychiatric illness	Yes: 8 No: 96	12.00 3.59	3.59 4.83	0.189	12.13 8.86	4.42 4.77	0.065	56.88 43.90	16.39 11.87	0.061	32.38 34.64	10.446 7.931	0.452
Parental consanguinity	Yes: 30 No: 74	10.17 9.74	4.39 4.95	0.684	9.50 8.96	3.69 5.20	0.552	47.80 43.72	11.90 12.85	0.137	33.70 34.77	7.74 8.29	0.545
Family history of death in early infancy	Yes: 33 No: 71	10.09 9.76	4.03 5.11	0.744	9.85 8.77	4.21 5.05	0.291	47.97 43.46	11.94 12.81	0.091	33.39 34.96	7.65 8.32	0.363
Employment status	Unemployed: 52 Working: 36 Retired: 16	10.10 9.92 9.00	5.02 4.66 4.40	0.726	8.98 8.97 9.88	5.25 4.51 4.08	0.793	47.54 41.28 44.44	13.78 11.34 9.96	0.072	33.29 36.75 33.13	8.430 8.436 4.992	0.111
Sex	Female: 60 (57.7%) Male: 44 (42.3%)	9.92 9.80	4.75 4.86	0.899	8.88 9.43	5.34 4.00	0.551	44.05 46.05	14.37 9.92	0.430	35.17 33.50	8.120 8.094	0.303
Education	Primary: 19 Secondary: 32 University: 53	8.26 10.50 10.06	4.98 4.85 4.62	0.250	9.74 9.22 8.83	4.15 5.50 4.64	0.775	46.53 45.44 43.98	10.35 12.27 13.74	0.726	32.95 32.56 36.15	5.307 9.442 7.841	.094
Income level	Low: 41 Adequate: 55 High: 8	**11.02** **8.62** 12.50	**4.97** **4.49** 3.30	**0.012**	**10.49** **8.02** 9.63	**5.29** **4.24** 4.53	**0.041**	**49.54** **42.25** 39.25	**12.47** **11.79** 13.44	**0.008**	**31.10** **36.15** 40.13	**8.182** **7.377** 6.379	**0.001**
Hospitalization	Yes: 79 No: 25	10.11 9.08	5.01 3.94	0.348	9.39 8.24	5.14 3.50	0.298	44.90 44.88	13.29 10.69	0.995	34.14 35.48	8.52 6.72	0.474
Siblings	Yes: 96 No: 8	9.65 12.50	4.73 4.87	0.105	9.01 10.38	4.61 6.97	0.602	44.82 45.75	12.29 17.50	0.843	34.59 32.88	7.52 14.06	0.742
Marital Status	Single: 35 Married: 58 Divorced: 11	10.17 9.38 11.45	4.48 4.63 6.33	0.378	8.97 8.69 11.82	4.74 4.67 5.27	0.137	46.91 44.36 41.27	15.13 11.15 11.59	0.392	35.51 34.09 33.09	7.946 8.327 7.816	0.603
Satisfaction with IGRT	Yes: 85 No: 15	9.28 12.60	4.36 6.08	0.059	8.49 **11.93**	4.68 **4.42**	**0.010**	43.42 **50.93**	12.49 **11.20**	**0.032**	34.84 31.80	8.08 8.60	0.187
Applied for disability report	Yes: 50 No: 53	**10.96** 8.98	**4.84** 4.46	**0.033**	**10.24** 8.11	**5.20** 4.22	**0.025**	**47.94** 41.94	**12.08** 12.72	**0.016**	**31.88** **36.96**	**8.06** **7.49**	**0.001**
Diagnosed psychiatric illness	Yes: 27 No: 76	10.04 9.66	4.47 4.76	0.719	9.59 8.80	5.02 4.60	0.456	42.33 45.59	12.68 12.57	0.251	33.96 34.93	8.75 7.54	0.583
Transportation	Public transport: 59 Private vehicles: 44	10.61 9.05	4.79 4.55	0.097	9.90 8.14	4.77 4.74	0.066	46.19 43.07	12.87 12.43	0.220	**32.95** 36.57	**8.22** 7.65	**0.025**
Place of residence	Village: 25	11.16	4.69	**0.004**	9.92	5.02	0.290	48.28	15.72	**0.012**	34.28	8.473	0.942
	Small town: 41	10.93	4.33		9.51	4.80		47.27	10.73		34.80	9.269	
	City: 38	**7.87**	**4.74**		8.16	4.63		**40.11**	**11.14**		34.21	6.597	

SASS: Social Adaptation Self-Evaluation Scale, HADS-A: Hospital Anxiety and Depression Scale–Anxiety, HADS-D: Hospital Anxiety and Depression Scale–Depression, and UCLA-LS: University of California Los Angeles Loneliness Scale

The proportion of participants who reported using public transportation was 75% (n = 30) among those with low income, 45.5% (n = 25) for those with adequate income, and 50% (n = 4) among those with high income. This rate was significantly higher for participants with low income (p = 0.014). Social functioning was highly impaired in public transportation users (p = 0.025). HADS-A, HADS-D, and UCLA-LS increased with length of hospital stay (r = 0.408, p < 0.01; r = 0.383, p = 0.001; and r = 0.278, p = 0.017, respectively). HADS-A was negatively correlated with age at diagnosis and symptom onset (r = -0.224, p = 0.022 and r = -0.271, p = 0.006, respectively).

## Discussion and Conclusion

This study is the first to assess SASS and UCLA-LS scales in adult PID patients, exploring their connections with HADS-A/HADS-D. Our findings revealed impaired social functioning, loneliness, and depressive symptoms in adult PID patients. Additionally, social maladjustment, anxiety, depression, and loneliness exhibited positive correlations within this population. All variables of interest were adversely affected in those who had applied for a disability report or had low income. Risk factors for loneliness included a family history of PID, comorbidities, prolonged hospital stay, dissatisfaction with IgRT, and living in a village or small town, while anxiety risk factors comprised residing outside the city and being younger at symptom onset and PID diagnosis. Depression risk factors involved prolonged hospital stay and dissatisfaction with IgRT.

Our study determined the risk of anxiety disorder as 44.2% and the risk of depression as 61.5% based on HADS-A and HADS-D scores; this aligns with trends observed in other studies using the Hamilton Anxiety Rating Scale [20]. Similarly, in a study where depression and anxiety risk were assessed in 96 patients diagnosed with common variable immunodeficiency using a variety of scales, including a generic, non-disease-specific instrument (SF-36) and the General Health Questionnaire (GHQ-12), approximately one-third of the patients were identified to be at risk of anxiety and depression during the observation period [11]. This finding suggests a consistent psychiatric profile among PID patients, despite the use of different measurement scales. Additionally, in a study with PID-diagnosed children, 70.45% exhibited psychiatric problems, with depression (27.3%), disruptive behavior disorders (27.3%), and anxiety disorders (18.2%) being the most prevalent diagnoses [21]. Another pediatric study indicated that clinically significant anxiety or depressive symptoms were observed in almost one out of every 4–5 children with PID [10]. As a result, anxiety and depression symptoms appear to be significantly high in PID patients, independent of screening tools and age groups. However, as observed in our study and many others, self-report measurements are commonly used instead of structured clinical interviews and criteria for anxiety and depressive disorders. This tendency may lead to higher prevalence estimates than those reported in studies involving clinical reassessments [22]. For these reasons, following the confirmation of PID diagnosis, it is recommended that instead of initiating anxiety and depression risk screening, patients undergo a psychiatric evaluation by a mental health specialist. This approach allows for early psychiatric monitoring and treatment initiation. However, its practicality and feasibility may pose challenges. Therefore, for a more comprehensive understanding and the development of strategies, further studies are needed, not only in the context of PID patients but also across chronic diseases in general.

Adults with immunodeficiency cannot work in physically demanding jobs or crowded environments and are often not preferred by employers due to their illness. Working patients, on the other hand, may have difficulty in taking leave for treatment and follow-up, and constantly feel the pressure of possibly being fired due to frequent hospital admissions. Despite the generally high educational levels among our patients (2.9% with no education or illiteracy, and 51% being university graduates), the 43.3% unemployment rate can be partially considered a result of this situation. Moreover, essential needs such as medication, medical examinations, and hospital transportation expenses can create a significant financial burden, contributing to the disruption of the balance between income and expenses, as observed in our patients.

In our study, patients who lived outside urban areas felt lonelier and more anxious. This finding is likely because cities offer better access to health care and more job and social opportunities. However, the cost of living is higher in cities than in rural areas. Moreover, we noted that patients using public transportation had poorer social functioning in our study group. When we investigated the characteristics of this subset of patients, we determined that most of them reported low income. In this study, the common problems of anxiety, depression, loneliness, and poor social functioning in PID patients seem to be related to financial distress. The literature confirms that individuals with chronic diseases and low socioeconomic status are at greater risk of limitation, dependency, social isolation, psychological distress, and impaired quality of life [16]. Living with PID in middle- and low-income countries is much more difficult and can lead to life-threatening problems. In our country, a disability health board report is required in order to benefit from the financial and social assistance provided by the state and private sector to disabled persons [23]. In our sample, 48.5% of the patients had applied for this report and 62% of those who applied were approved (30% of all patients). However, this assistance should be expanded to cover basic needs, medical costs, and social needs for PID patients. In addition, introducing new criteria for disability reports for PID patients or making the process of getting the report easier should be considered.

In recent years, it has been clearly seen that switching to at-home SCIG treatment instead of hospital-based IVIG has increased the quality of life [24, 25]. In our patients, rates of satisfaction with IGRT were found to be highest in those receiving IVIG (88%), followed by SCIG (76.2%) and fSCIG (66.7%), with no significant difference in satisfaction between the different infusion methods. This result may be because all infusion options are evaluated jointly with the patients in our clinic. Treatment is selected according to the patients’ needs and conditions and subsequently revised according to their satisfaction and compliance with the treatment. Despite this, 15% of our patients were not satisfied with IGRT, and symptoms of depression and loneliness were more common in these patients. Moreover, we observed in this study that younger age at diagnosis and symptom onset were associated with higher anxiety, while anxiety, loneliness, and depression increased with longer hospital stays. When our patients were evaluated overall, their disease and treatment history significantly affected their psychosocial status. Different studies investigating different parameters have demonstrated a relationship between depression and severe disease, frequent hospital admissions, a high number of complications, a high number of annual infections, not being able to come to the hospital by car, IVIG (vs. SCIG) treatment, nurse-administered treatment (vs. self-administered), contralateral side effects of IGRT, and suicide attempts. On the other hand, poor health status, unhealthy diet, and lack of restful sleep were reported as risk factors for anxiety [21, 26]. In addition, delay in diagnosis for more than six years and the presence of a family history of anxiety and/or depression have been shown to have a significant effect on both anxiety and depression scores [20, 26].

Franco et al. conducted a behavioral and neuroanatomical evaluation of the effects of loneliness and social adjustment on depressive symptoms. They showed that loneliness enhances depressive symptoms but has a positive effect on social functioning. Additionally, loneliness, social functioning, and depressive symptoms share a common white matter area in the brain [27]. A five-year longitudinal study provided evidence of a unilateral relationship in which loneliness predicted, if not promoted, increases in depressive symptomatology in middle-aged and older adults. The authors also noted that this temporal relationship could not be attributed to demographic variables, objective social isolation, temperamental negativity, stress, or social support [12]. However, in a longitudinal cohort study including older adults, loneliness was an important predictor for depression but showed no significant association with anxiety [13]. In addition, Saris et al. suggested that social disability acted as a predictor of anxiety and/or depressive disorders (8). In the current study, social maladjustment, anxiety, depression, and loneliness were found to correlate with each other. However, since our study was cross-sectional, we cannot determine the causality of these relationships.

In conclusion, social dysfunction, loneliness, anxiety, and depression are common in adults with PID. The results of this study highlight the need to evaluate these patients in terms of their psychiatric, social, and economic needs with an interdisciplinary team including clinicians, social workers, and psychiatrists.

## Data Availability

Availability of data and materials: The authors confrm that the data supporting the fndings of this study are available within the article and/or its supplementary materials. If you have questions regarding the data, contact the corresponding author.
